# Case study: Evaluating deep-water start techniques and training demands in seated slalom waterskiing for an athlete with paraplegia

**DOI:** 10.3389/fpsyg.2024.1363544

**Published:** 2024-06-19

**Authors:** David Suárez-Iglesias, Carlos Ayán, Adrián García-Fresneda, José Gerardo Villa-Vicente, Juan Rodríguez-Medina, Jose A. Rodríguez-Marroyo

**Affiliations:** ^1^VALFIS Research Group, Institute of Biomedicine (IBIOMED), Universidad de León, León, Spain; ^2^Faculty of Physical Activity and Sports Sciences, Universidad de León, León, Spain; ^3^Well-Move Research Group, Department of Special Didactics, Faculty of Education and Sport Science, Galicia Sur Health Research Institute (IIS Galicia Sur), Universidad de Vigo, Pontevedra, Spain; ^4^Department of Health Sciences, Research Group in Technology Applied to High Performance and Health, TecnoCampus, Universitat Pompeu Fabra, Mataró, Barcelona, Spain

**Keywords:** water sports, disability impairment, heart rate, perceived exertion, training zones

## Abstract

**Purpose:**

Recreational and competitive slalom waterskiing is increasingly popular among individuals with spinal cord injuries (SCI), particularly for those with paraplegia using sit-skis. A key component of slalom skiing is the deep-water start (DWS), yet little is known about the physiological and physical demands of this activity when the athlete is seated. This study aims to fill this gap by focusing on the training requirements for a seated slalom athlete.

**Materials and methods:**

Focusing on a young male athlete with paraplegia, this case study evaluates the effectiveness and efficiency of traditional (TDWS) and alternative (ADWS) DWS techniques during seated slalom waterskiing sessions. It assesses internal training load (TL) through heart rate (HR) zones and session rating of perceived exertion (sRPE), alongside pre- and post-session handgrip strength measurements to gauge peripheral muscle fatigue.

**Results and conclusions:**

Performing the ADWS, achieving a full success rate, proved more effective but slightly more time-consuming than TDWS, which had limited success. HR during DWS maneuvers ranged from 63.2 to 81.3% of maximal HR, with most sessions occurring below the ventilatory threshold, thus perceived as hard effort. A moderate yet non-significant correlation was found between HR and sRPE-based TL. A significant reduction in handgrip strength post-session underscores the activity’s demands. These insights illuminate the technical, physiological, and physical challenges in mastering DWS for seated slalom athletes with SCI, providing valuable guidance for the development of tailored training programs and techniques in this sport.

## 1 Introduction

In recent decades, Para athletes have gained greater prominence and have increasingly participated in a wide range of events (Baumgart et al., 2022). Therefore, there have been growing concerns about the necessity for advanced physical fitness and comprehensive training technique knowledge to optimize performance in athletes with physical impairments (Gee et al., 2021; Rodríguez Macías et al., 2022). Although contemporary studies have explored training methods for wheelchair sports (Simim et al., 2017; Baumgart et al., 2018), research has remained limited on competitive sporting disciplines outside the context of the Paralympic Games (Lexell and Frontera, 2023).

In this regard, waterskiing, a popular towed water sport activity, has provided leisure opportunities for individuals with spinal cord injury (SCI) (Urbański et al., 2021); and caters to various ability levels, including those who cannot stand (Suárez-Iglesias and Villa-Vicente, 2017). In the discipline of slalom, whether for athletes in the seated division or those able to stand, success hinges on mastering a repeatable technique that reduces load during the deep-water start (DWS) and on executing DWS with precision (Bray-Miners et al., 2012). This process entails navigating a sharp increase in tow rope tension, which can surge to 2.0–2.5 times an athlete’s body weight (Keverline et al., 2003; Runciman, 2011). Lighter athletes may face even higher relative peak tensions, with skill level additionally influencing this tension (Suderman et al., 2023; Lance, 2024). Overcoming this significant initial tension requires considerable upper-body strength and endurance, especially for seated slalom athletes who primarily rely on their upper-body muscles to transition from water to a seated skiing position (Suárez-Iglesias et al., 2019b). To mitigate grip force and fatigue that may arise from maintaining the connection to the boat and control of tension on the line (Woodgate et al., 2021), various DWS methods are employed, including instructor-assisted starts and specific learning aids (USA Water Ski Level 1 Instructor’s Manual, 2012).

However, research on the experiences of seated slalom athletes with the DWS has been limited. For the development of an effective and efficient DWS, incorporating sport science principles that address the physiological processes activated during exercise is crucial (Hoffmann et al., 2014). Collecting data on the physical workload of seated slalom athletes may potentially enhance coaches’ ability to individualize training programs. This targeted approach could lead to more precise management of an athlete’s physical workload, potentially aiding in the effective monitoring and development of the athlete. Such an approach might also contribute to improved sports performance and could possibly reduce the risk of injuries and illnesses (Stieler et al., 2023).

In this context, recent studies have specifically investigated the experiences and physiological impacts on athletes engaged in seated slalom waterskiing. The first study, involving three males with paraplegia, found that recreational seated slalom waterskiing generally entailed moderate intensity, with an average heart rate (HR) reserve around 45% (Suárez-Iglesias et al., 2019a). The second study engaged five adults from a rehabilitation center with various physical impairments in a beginner’s seated slalom waterskiing course, and explored participants’ perceptions of wellness, learning, and enjoyment. It reported that session intensity varied from “fairly light” to “somewhat hard” on the original Borg Rating of Perceived Exertion (RPE) scale 6–20 (Suárez-Iglesias and Villa-Vicente, 2017). While these studies employed HR and RPE metrics— common tools in athletic performance monitoring for wheelchair sports (Simim et al., 2017) —their focus diverged from typical performance evaluations in practices, where HR and RPE-based training zones are benchmarks for coaches to assess and monitor athletes’ seasonal progress (Rodríguez-Marroyo et al., 2012; Yanci et al., 2015). Instead, these studies have highlighted broader aspects, such as the health benefits and inclusivity of participating in seated waterskiing for leisure.

Furthermore, tailoring training to an athlete’s stress levels, as indicated by grip force, can enhance training outcomes, according to Sahar et al. (2022). Handgrip strength, a common indicator of physical performance in adaptive athletes (O’Connor et al., 2022), warrants close attention, particularly in seated slalom athletes. Supporting this observation, research including a study with four males at a national championship revealed that seated slalom waterskiing significantly reduced handgrip strength. Notably, the degree of this reduction varied according to each athlete’s level of competition and overall fitness (Suárez-Iglesias et al., 2019b). Consequently, these findings suggested that monitoring handgrip strength could be instrumental in customizing training programs to optimize the performance and wellbeing of seated slalom athletes.

In light of the scenario previously outlined, this case study addressed the need for specialized training programs and an in-depth understanding of the physiological demands in seated slalom waterskiing. This research is centered around a young male athlete with SCI. It is methodically structured with a threefold objective: first, to quantitatively evaluate the efficacy and efficiency of both traditional and alternative DWS methodologies tailored to his specific condition; second, to analyze his exertion levels using HR zones and session-RPE (sRPE) for a comprehensive assessment of TL; and third, to assess his peripheral muscle fatigue, emphasizing handgrip strength measurements.

## 2 Method

### 2.1 Participant

This case study involved a 28-year-old male athlete with T5 complete paraplegia (ASIA Impairment Scale A) (Kirshblum et al., 2011), resulting from a motor vehicle accident 7 years prior. He was 1.77 meters tall and weighed 55.0 kg. With 3 years of experience in seated slalom waterskiing, he primarily trained at a cable park and on a reservoir, adopting a position with his knees above his hips and using an 18.2-meter tow rope. Identified as an intermediate-advanced athlete (Runciman, 2011; Bray-Miners et al., 2012), he often found it challenging to get out of the water independently, which usually led to failed or exhausting attempts. To overcome this, he undertook training in a new DWS technique. He was fully briefed about the study and provided his informed consent. The study received ethical approval from the University of León, Spain, and was conducted in accordance with the Declaration of Helsinki.

### 2.2 Procedure

#### 2.2.1 Seated slalom waterskiing program

The program comprised six seated slalom waterskiing sessions, spanning three sessions per week over two consecutive weeks, focused on learning, practicing, and refining two DWS techniques. The traditional technique involved the athlete sitting in the water and being pulled up onto the surface by the towboat without assistance (Bray-Miners et al., 2012). In contrast, the assisted alternative employed “the boom” (a training bar) attached to the port side of the towboat (Kegel, 1985), with a short rope gradually extended to align the seated slalom athlete centrally behind the towboat.

Each session began with a standardized 5 min warm-up consisting of mobility exercises on the towboat, followed by the introduction of the alternative technique for water-based training. After successfully completing a DWS, the athlete engaged in typical activities, predominantly slalom runs (Suárez-Iglesias et al., 2019a). These runs alternated between open water and an adapted slalom course, referred to as the inner-slalom course, with buoys set 6.4 meters from the course axis (International Waterski & Wakeboard Federation, 2023). In the event of a fall, the athlete was required to perform a DWS and restart the practice, incorporating a rest period for coaching feedback. The program was conducted at a reservoir in Northern Spain.

Special precautions were taken for the participant due to his SCI at T5, which increased the risks of hypothermia and autonomic dysreflexia. These included: (a) emptying the bladder or urine collection bag before each session; (b) wearing a wetsuit as a preventative measure; (c) ensuring the participant had free access to fluids at all times; and (d) avoiding prolonged rest periods in the towboat with wet clothes (Willis et al., 2018). Weather conditions varied, with air temperatures ranging from 20 to 32°C, wind speeds from 12 to 37 km/h, and water temperatures between 14 and 16°C. Waterskiing occurred in sheltered reservoir areas to minimize wind exposure and ensure smoother water conditions, away from other boat wakes. The athlete wore a waterskiing vest for safety (Loughlin, 2013). To control for variables that might affect outcomes, all sessions were conducted at similar times from 11:45 a.m. to 2:15 p.m., using the same towboat and experienced driver, with speed controlled by PerfectPass (PerfectPass Control Systems Inc., Dartmouth, NS, Canada), on the same inner-slalom course, and without altering the tow rope length.

#### 2.2.2 Data collection

##### 2.2.2.1 Content of seated slalom waterskiing sessions

**Figure 1:**
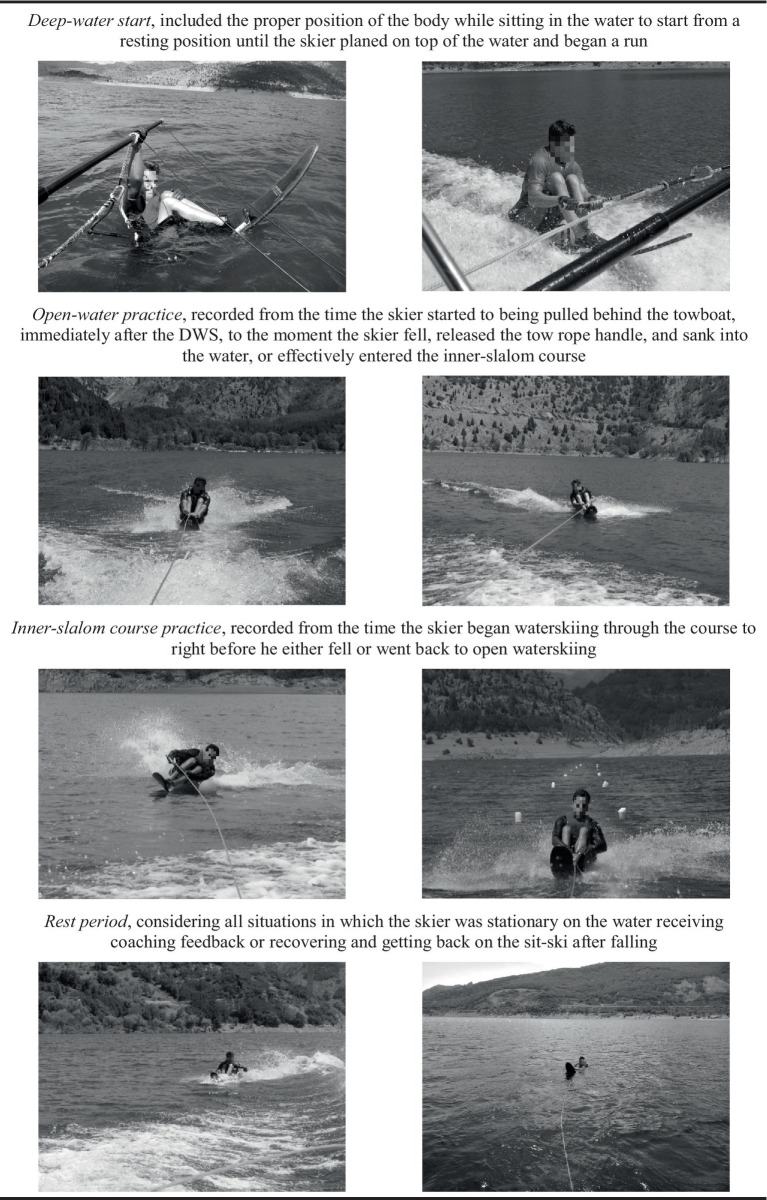
Main activities during seated slalom waterskiing sessions.

An expert observer quantified characteristics and events during the sessions, making detailed written records of their nature, duration, and towboat speeds (Traceable manual digital chronometer VWR, Pennsylvania, United States). Activities were categorized into four broad groups based on their nature (see Figure 1). Within the *deep-water start* category, further differentiation was made to distinguish between two phases, when the seated slalom athlete performed *alternative* (ADWS) and *traditional* (TDWS) techniques (for further details, see Supplementary material).

##### 2.2.2.2 Effectiveness and efficiency of deep-water starts

The same expert recorded the number of attempted ADWS and TDWS maneuvers. To evaluate effectiveness, the number of errors committed during these maneuvers was noted, while efficiency was assessed based on the time taken to complete each maneuver (Mohanraj et al., 2023). In terms of effectiveness, errors were categorized as falls, in line with the official rules for a seated slalom athlete (International Waterski & Wakeboard Federation, 2023). A fall is defined as occurring when the athlete: (a) loses possession of the tow line; (b) fails to maintain possession of the skiing device; or (c) is not predominantly supported by the skiing device and cannot regain a seated position. Conversely, a successful DWS attempt is characterized by the athlete adopting a seated position, which includes maintaining possession of the tow line, riding forward or backward on the skiing device, and having their weight fully supported by the skiing device or being able to ultimately regain control. For efficiency analysis, the time from the start of a successful ADWS or TDWS attempt until the athlete achieves a seated position was measured.

##### 2.2.2.3 Internal training load

The exercise demands were quantified based on HR and RPE (Foster et al., 2001; Rodríguez-Marroyo et al., 2012). Heart rate was recorded every 5 s during each training session using the Polar Team System 2 (Polar Electro Oy, Kempele, Finland). After the sessions, HR data were downloaded to a computer using specific software (Polar Pro Trainer 5, Polar Electro Oy, Kempele, Finland). The participant’s cardiorespiratory fitness was evaluated via a laboratory test performed prior to the start of the seated slalom waterskiing program (Suárez-Iglesias et al., 2019a). Based on the HR values obtained from this test, HR responses were categorized into three distinct intensity zones. These zones were defined in relation to the ventilatory threshold (VT) and respiratory compensation threshold (RCT): zone 1 (low-intensity exercise) was below VT; zone 2 (moderate-intensity exercise) was between VT and RCT; and zone 3 (high-intensity exercise) was above RCT (Rodríguez-Marroyo et al., 2012). These zones were used to calculate the TL by multiplying the time spent in zones 1, 2, and 3 by the constants 1, 2, and 3, respectively. The TL score was obtained by summing the results of the three phases. The intensity of seated slalom waterskiing sessions was evaluated using Borg’s category-ratio (0–10) RPE scale, known as Borg-CR10; collected approximately 30 min after each training session (Foster et al., 2001). The participant was already familiar with this scale, having used it routinely to control training intensity during the year prior to the study. The sessions were categorized into three intensity levels based on the RPE values: moderate (RPE < 5), hard (RPE 5–6), and fairly hard (RPE > 6) (Rodríguez-Marroyo and Antoñan, 2015). Moreover, the TL was calculated using the sRPE, by multiplying the RPE value by the duration of the training session (Foster et al., 2001).

##### 2.2.2.4 Handgrip strength

Handgrip strength was measured using a digital dynamometer (Takei TKK 5401 Grip-D, Tokyo, Japan) immediately before and after each seated slalom waterskiing session. The participant adopted a standardized testing position according to the guidelines of the American Society of Hand Therapists (ASHT), sitting in the towboat with the elbow bent at 90 degrees (Sisto and Dyson-Hudson, 2007). The participant selected the handgrip position on the dynamometer that allowed for maximum force exertion (Boadella et al., 2005). Peak force was recorded in kilograms over a 5 s period; three consecutive maximal repetitions were conducted for the dominant hand, with 60 s rest intervals between each repetition. The highest value from the three repetitions was used for analysis. The strength decrement index for each session was calculated as follows: 100% × (Initial Max – Final Max) / Initial Max (Reuter et al., 2011).

All measurements were conducted by the same researcher, who was experienced in the procedures for evaluating physical fitness in individuals with physical impairments.

#### 2.2.3 Statistical analysis

Data were presented as mean ± standard deviation (SD). Normality was assessed using the Shapiro–Wilk test. The relationship between HR-based and sRPE-based TL methods was determined using Pearson’s correlation coefficient (*r*). The magnitude of the correlation was classified as: trivial (< 0.1), small (0.1–0.3), moderate (0.3–0.5), large (0.5–0.7), very large (0.7–0.9), and nearly perfect (> 0.9) (Hopkins, 2002). A *p*-value of <0.05 was considered statistically significant. Pre- and post-session handgrip strength values were compared using a paired student’s t-test. Statistical analyses were performed using SPSS version 24.0 (Chicago, Illinois, United States).

## 3 Results

### 3.1 Content of seated slalom waterskiing sessions

Key characteristics of the seated slalom waterskiing sessions are summarized in Table 1. The average session duration was 30 ± 7 min. The ADWS phase constituted 8.4 ± 5.2% of the total session time, TDWS 5.5 ± 4.4%, *open-water practice* 48.3 ± 23.6%, *inner-slalom course practice* 16.3 ± 16.5%, and *rest periods* 21.5 ± 13.7%. Towboat speeds ranged from 31 to 43 km/h.

**Table 1:** Summary of data for the seated slalom waterskiing sessions.

Seated slalom waterskiing session	1	2	3	4	5	6
**Total duration (min:s)**	34:33	27:43	29:50	40:46	28:31	20:00
Alternative deep-water start	03:19	01:26	05:10	04:16	01:05	00:50
Traditional deep-water start	03:25	–	02:46	00:55	02:37	00:30
Open-water practice	17:05	26:17	11:31	14:29	08:47	08:05
Inner-slalom course practice	03:44	–	–	06:20	08:58	08:00
Rest period	07:00	–	10:23	14:46	07:04	02:35
**Towboat speed (km·h** ^**−1**^ **)**	31	34	34	34	34–43	37–40
**Alternative deep-water start**						
Number of attempts	2	1	5	3	1	1
Number of successful attempts	2	1	5	3	1	1
**Traditional deep-water start**						
Number of attempts	2	–	2	2	3	1
Number of successful attempts	0	–	0	2	0	0

### 3.2 Effectiveness and efficiency of deep-water starts

During the 6-day seated slalom waterskiing program, the participant executed a total of 23 DWS, comprising 13 ADWS attempts and 10 TDWS attempts (Table 1). In terms of effectiveness, the ADWS method achieved a 100% success rate, with each attempt resulting in the athlete adopting a seated position. In contrast, the TDWS method had a lower success rate of 20%. Regarding efficiency, the ADWS method averaged 74 s per attempt, totaling 16 min and 6 s, while the TDWS method was quicker, averaging 61 s per attempt, totaling 10 min and 13 s.

### 3.3 Internal training load

Physiological demands experienced by the seated slalom athlete are detailed in Table 2. Analyzed as percentages of maximal HR (%HR_max_), the average HR during ADWS phases was 70.8 ± 6.3% HR_max_, and during TDWS phases, it was 69.6 ± 4.5% HR_max_. The overall average intensity across all seated slalom waterskiing sessions was 71.8 ± 4.4% HR_max_. Time distribution across the intensity zones was 76.7 ± 15.3% in zone 1, 21.7 ± 14.2% in zone 2, and 1.7 ± 1.6% in zone 3. The mean RPE was 4.7 ± 1.6. The mean TL, assessed using HR and sRPE, was 39.6 ± 3.1 and 151.6 ± 61.1 arbitrary units (AU), respectively. A moderate positive correlation (*r* = 0.45, *p* = 0.37) between HR-based TL and sRPE-based TL was found, though it was not statistically significant.

**Table 2:** Percentage of maximal heart rate during deep-water starts and percentage time spent in each training zone, session rating of perceived exertion, training load, pre- and post-training values on handgrip strength measurements and Strength Decrement Index during the seated slalom waterskiing sessions.

Training session	1	2	3	4	5	6
**Percentage of maximal HR**						
Alternative deep-water start	75.3	81.3	68.1	64.3	68.1	67.6
Traditional deep-water start	70.9	-	75.3	63.2	67.6	70.9
**Time spent in the training zones**						
Zone 1 (%)	82	55	83	99	76	65
Zone 2 (%)	16	41	17	1	21	34
Zone 3 (%)	2	4	0	0	3	1
**RPE (Borg-CR10 score)**	5.0	3.5	2.0	5.5	6.5	5.5
**sRPE-based TL (AU)**	172.8	97.0	59.7	224.2	185.4	170.5
**HR-based TL (AU)**	41.5	41.3	34.9	41.2	36.2	42.2
**Handgrip strength**						
Pre (kg)	49.1	52.0	50.7	51.8	46.9	48.3
Post (kg)	36.5	49.3	48.5	40.4	43.1	44.6
Strength decrement index (%)	25.7	5.2	4.3	22.0	8.1	7.7

Borg-CR10, category-ratio (0–10) RPE scale; RPE, rating of perceived exertion; sRPE, session rating of perceived exertion; HR, heart rate; TL, training load; AU, arbitrary units; Pre, before training; Post, after training.

### 3.4 Handgrip strength

Pre- and post-session handgrip strength values are presented in Table 2. The mean post-session value (43.7 ± 4.9 kg) was significantly lower than the pre-session value (49.8 ± 2.0 kg) (*p* = 0.024). The mean strength decrement index, indicating the proportionate deterioration from pre-session values, was 12.2 ± 9.2%. Strength decrement index values exceeded 20% in the first and fourth seated slalom waterskiing sessions.

## 4 Discussion

This case study contributes valuable perspectives on seated slalom waterskiing training for athletes with physical impairments, specifically focusing on an individual with paraplegia. It explores the potential effectiveness and efficiency of various DWS techniques, both traditional and alternative. Additionally, the study assesses impacts on internal TL, and handgrip strength during DWS and ongoing seated slalom activities.

In waterskiing, which demands considerable physical fitness and advanced skills (Mullins, 2007; Woodgate et al., 2021), the current study’s comparison of ADWS versus TDWS offers insightful observations, especially for coaches and athletes with SCI in seated slalom waterskiing programs. The analysis of effectiveness and efficiency suggests that ADWS, achieving a 100% success rate, may provide greater reliability and control – essential in this complex maneuver, especially when using adaptive equipment in extreme sports (Allen et al., 2021). Conversely, despite being faster, TDWS shows a lower success rate of 20%, potentially leading to athlete frustration, interruption of practice continuity, and a heightened risk of energy depletion and injury. These findings could inform training strategies that prioritize a balance between performance, safety, and technical skill (Gunderson, 1991; Suárez-Iglesias et al., 2019b), while acknowledging the sport’s challenging learning curve, the need for mastery over specialized equipment, adaptability to various environments, and the diverse components and time management within both standing and seated slalom waterskiing sessions (Thye and Rokosz, 1991; Bray-Miners et al., 2015; Suárez-Iglesias et al., 2019a).

Regarding HR monitoring for gauging exercise intensity, the current research found the participant primarily engaged in aerobic metabolism during seated slalom waterskiing sessions, evident from the predominant time spent in HR zones 1 and 2, similar to standing slalom waterskiing (Mullins, 2007) and paralleling findings in Para alpine skiing (Goll et al., 2015). Such data suggest moderate physical strain in seated slalom waterskiing, consistent with prior research in this discipline (Suárez-Iglesias et al., 2019a). This pattern can likely be attributed to the intermittent nature of the sessions, which, in both previous and current studies, involve seated slalom waterskiing sessions exceeding 10 min but interspersed with *rest periods* and varied training components, such as *open-water practice* and *inner-slalom course practice*.

In addition, this research records for the first time %HR_max_ values during DWS, ranging from 63.2 to 81.3%. These reflect slalom waterskiing’s high static and low dynamic demands (Mitchell et al., 2005) and are comparable to HR patterns reported in Para alpine skiing athletes (Goll et al., 2015). The comparable short duration of these activities, akin to the 55 s Para alpine skiing giant slalom runs, may explain these figures. Moreover, the similarity in HR responses between the seated slalom athlete during DWS and athletes in wheelchair tennis or basketball (Roy et al., 2006; Barfield et al., 2009; Croft et al., 2010; Sindall et al., 2013) further highlights the shared intermittent nature of these sports.

This investigation is the first to explore RPE in seated slalom waterskiing. The findings showed that the intensity of seated slalom waterskiing sessions ranged from moderate to fairly hard, according to the RPE categories defined by Rodríguez-Marroyo and Antoñan (2015). These RPE values, exceeding 5 on several occasions, suggest that the participant found the activity to be quite challenging. This contrasts with a study on elite wheelchair tennis athletes who reported lower RPE during singles matches (Sánchez-Pay et al., 2016), possibly due to differences in skill and experience levels. Evidence in the literature supports these findings, showing that perceived exertion can be influenced by skill level, as seen in recreational alpine skiers with advanced abilities reporting lower exertion (Scheiber et al., 2009).

It is now well established that TL monitoring is crucial for understanding an athlete’s adaptation to training and assisting coaches in program design (Halson, 2014; Simim et al., 2017; Rodríguez Macías et al., 2022; Stieler et al., 2023). The present study revealed differing TL scores when using HR and sRPE-based methods. The TL values obtained in this study were lower than those reported for wheelchair basketball athletes during small-sided games (55.3–67.5 AU) (Iturricastillo et al., 2017) and notably less than TL reported in elite wheelchair rugby athletes (247 ± 74 AU) using the same HR-based method (Paulson et al., 2015). These differences may stem from the distinct intensities and activities in wheelchair sports, characterized by high-intensity, intermittent wheelchair use, and varying eligible impairments (with wheelchair basketball encompassing diverse physical impairments and wheelchair rugby often involving athletes with cervical SCI). The sRPE-based TL values from this study were higher than those in wheelchair basketball for small-sided games (99.3 ± 26.9 AU) (Iturricastillo et al., 2017) but lower than in wheelchair rugby practice (934 ± 359 AU) (Paulson et al., 2015). These variances emphasize the need for cautious interpretation, considering differences in participant characteristics, activities, and training session durations across studies.

The intersection of objective and subjective measures in TL assessment for athletes with physical impairments is a topic warranting further exploration. The current research noted a moderate, yet non-significant, correlation between HR and sRPE-based TL (*r* = 0.45; *p* = 0.37) underscores the nuanced nature of seated slalom waterskiing’s static demands, particularly for individuals with paraplegia. In such instances, HR typically shows modest increases during fatiguing isometric exercises, a response linked to central factors (Petrofsky, 2001). Besides, the sRPE scores in this study, encompassing both central and peripheral inputs (Borg, 1982), might have been influenced by these factors. Firstly, the repetitive nature of DWS and the prolonged duration of seated slalom waterskiing sessions, often surpassing the typical 15 min waterskiing times on slalom courses (Mullins, 2007), could have resulted in heightened central fatigue in the participant. Secondly, the requirement for meticulous force adjustments and reactions to external disturbances in seated slalom waterskiing could have additionally contributed to peripheral muscle fatigue (Ferguson, 2010). The findings of this study are consistent with research indicating small or trivial correlations between HR and sRPE-based TL in wheelchair basketball (range *r* = − 0.30; ± 0.27 to 0.26; ± 0.28; *p* > 0.05) (Iturricastillo et al., 2017) yet they diverge from studies showing stronger correlations in wheelchair basketball matches (*r* = 0.63–0.65, *p* < 0.001) (Iturricastillo et al., 2016) and wheelchair rugby training sessions (*r* = 0.81) (Paulson et al., 2015). These variations might arise from differences in functional abilities among participants or diverse settings (e.g., small-sided games, training sessions, matches) in different studies.

The present research observed a significant decline in handgrip strength post-session, likely due to the intense engagement of finger flexors and forearm extensors required in tow rope handling (Mullins, 2007). This finding echoes a previous study (Suárez-Iglesias et al., 2019b), which showed an 18.5% average decrease in maximum handgrip strength among four seated slalom waterskiing athletes with SCI after 14 waterskiing sessions, typically after about 20.5 min of grip time. Comparable reductions are noted in judo, which similarly demands extensive use of these muscles (Bonitch-Góngor et al., 2012). In slalom waterskiing, gripping technique also influences forearm muscle load (Rosa et al., 2016); thus, we recommend coaches prioritize optimal upper limb positioning to improve neuromuscular performance in seated slalom waterskiing. Specifically, the ADWS technique, starting with a parallel grip on “the boom,” may offer advantages over TDWS tow rope handling from the outset of the maneuver. Future research should encompass a broader group of athletes across multiple seated slalom waterskiing sessions, focusing on a detailed comparison of grip strength pre- and post-successful DWS using both ADWS and TDWS.

Despite its contributions to research for sports for people with physical impairments (Liu et al., 2022), the study is not without limitations. Primarily, the reliance on a single participant restricts the generalizability of the findings to the broader population of athletes with SCI (Rayes et al., 2022). It is important to acknowledge that the limited participation of athletes with SCI in research inherently restricts the breadth of training recommendations, which often rely on the anecdotal experiences of coaches, athletes, and scientists (Perret, 2017). Additionally, the lack of a time-motion analysis, which would provide external training load metrics like distance, speed, and duration (Stieler et al., 2023) limits a more holistic understanding of the participant’s TL.

For intermediate-advanced seated slalom athletes with paraplegia, initially focusing on ADWS, despite being slightly more time-consuming than TDWS, provides a reliable foundation that leads to a higher number of successful water exits. This allows coaches to allocate more time to other essential waterskiing skills, such as those typically practiced during open-water skiing and navigating the inner-slalom course (e.g., crossing the wake and making precise turns). Once athletes are consistently successful with ADWS, coaches can gradually incorporate TDWS into their training sessions, as it is the DWS required for sanctioned events. This balanced approach, involving both DWS techniques and comprehensive training in all technical aspects of waterskiing, facilitates continuous and holistic athlete progression towards the competitive level.

Future research should investigate the long-term effects of integrating ADWS before transitioning to TDWS on seated slalom athletes with paraplegia. Evaluating key performance indicators such as skill acquisition speed, maneuver completion rates, athlete confidence, and injury incidence can provide valuable insights. This research could optimize training protocols, enhancing both the efficiency and safety of athlete development in competitive seated slalom waterskiing.

In conclusion, this study sheds new light on seated slalom waterskiing by comparing two DWS techniques for an athlete with SCI. The findings suggest that ADWS, with its full success rate, appears more effective but slightly more time-consuming than TDWS, which presented more challenges and fewer successes for the participant. The moderate TL during seated slalom waterskiing was perceived as a hard effort by the athlete with paraplegia, and the significant reduction in handgrip strength post-session underscores the discipline’s grip strength requirements. These insights enrich understanding of the technical, physiological, and physical demands in mastering DWS in seated slalom waterskiing, offering valuable guidance for coaches and athletes in creating specialized training strategies and techniques.

## Data availability statement

The raw data supporting the conclusions of this article will be made available by the authors, without undue reservation.

## Ethics statement

The studies involving humans were approved by Comité de Ética de la Universidad de León. The studies were conducted in accordance with the local legislation and institutional requirements. The participants provided their written informed consent to participate in this study. Written informed consent was obtained from the individual(s) for the publication of any potentially identifiable images or data included in this article.

## Author contributions

DS-I: Conceptualization, Data curation, Funding acquisition, Investigation, Visualization, Writing – original draft, Writing – review & editing. CA: Supervision, Writing – original draft, Writing – review & editing. AG-F: Formal analysis, Writing – original draft, Writing – review & editing. JV-V: Conceptualization, Data curation, Funding acquisition, Methodology, Writing – original draft, Writing – review & editing. JuR-M: Writing – original draft, Writing – review & editing. JoR-M: Methodology, Project administration, Resources, Writing – original draft, Writing – review & editing.
